# Hydrogen Attenuates Endotoxin-Induced Lung Injury by Activating Thioredoxin 1 and Decreasing Tissue Factor Expression

**DOI:** 10.3389/fimmu.2021.625957

**Published:** 2021-03-09

**Authors:** Qian Li, Liang Hu, Juan Li, Pan Yu, Fan Hu, Bing Wan, Miaomiao Xu, Huixian Cheng, Wanyou Yu, Liping Jiang, Yadan Shi, Jincan Li, Manlin Duan, Yun Long, Wen-Tao Liu

**Affiliations:** ^1^Department of Anesthesiology, Jiangning Hospital Affiliated to Nanjing Medical University, Nanjing, China; ^2^Jiangsu Key Laboratory of Neurodegeneration, Department of Pharmacology, Nanjing Medical University, Nanjing, China; ^3^Department of Anesthesiology, Jinling College Affiliated to Nanjing Medical University, Nanjing, China; ^4^Department of Anesthesiology, Jinling Hospital, School of Medicine, Nanjing University, Nanjing, China; ^5^Department of Burn and Plastic Surgery, Jinling Hospital, School of Medicine, Nanjing University, Nanjing, China; ^6^Department of Anesthesiology, Jinling Hospital, The First School of Clinical Medicine, Southern Medical University, Nanjing, China; ^7^Department of Anesthesiology, Guangdong Provincial People's Hospital, Guangzhou, China

**Keywords:** hydrogen, sepsis, lung, Trx1, TF, MMP-9

## Abstract

Endotoxin-induced lung injury is one of the major causes of death induced by endotoxemia, however, few effective therapeutic options exist. Hydrogen inhalation has recently been shown to be an effective treatment for inflammatory lung injury, but the underlying mechanism is unknown. In the current study we aim to investigate how hydrogen attenuates endotoxin-induced lung injury and provide reference values for the clinical application of hydrogen. LPS was used to establish an endotoxin-induced lung injury mouse model. The survival rate and pulmonary pathologic changes were evaluated. THP-1 and HUVECC cells were cultured *in vitro*. The thioredoxin 1 (Trx1) inhibitor was used to evaluate the anti-inflammatory effects of hydrogen. Hydrogen significantly improved the survival rate of mice, reduced pulmonary edema and hemorrhage, infiltration of neutrophils, and IL-6 secretion. Inhalation of hydrogen decreased tissue factor (TF) expression and MMP-9 activity, while Trx1 expression was increased in the lungs and serum of endotoxemia mice. LPS-stimulated THP-1 and HUVEC-C cells *in vitro* and showed that hydrogen decreases TF expression and MMP-9 activity, which were abolished by the Trx1 inhibitor, PX12. Hydrogen attenuates endotoxin-induced lung injury by decreasing TF expression and MMP-9 activity *via* activating Trx1. Targeting Trx1 by hydrogen may be a potential treatment for endotoxin-induced lung injury.

## Introduction

Sepsis is related to systemic inflammatory response syndrome and results in dysfunction or organ failure with a mortality rate up to 40% while endotoxemia is one of the main pathogenic factors of sepsis (1). Acute lung injury often occurs in the early stage of endotoxemia and is one of the most important causes of death (2); effective specific treatments are lacking. Exploring the pathogenesis of endotoxemia and seeking effective prevention strategies are urgently needed.

Endotoxin-induced lung injury is mainly due to bacterial and viral infections, resulting in the excessive production of inflammatory cytokines within a short time, followed by inflammatory and coagulation cascade reactions (3). During this process, tissue factor (TF) plays a key role as an initiator of cascade reactions (4, 5). It has been reported that inhibition of TF significantly increases the survival rate during endotoxemia (6–8).

TF activation is mainly influenced by two processes. First, the cleavage of its activation site by upstream and downstream factors (9). Matrix metalloproteinase (MMP)-9 exerts the greatest effect on the hydrolytic cleavage of activated TF (10). MMP-9 not only affects TF activation, but also interacts with MMP-2 to deteriorate the respiratory tract, and degrade the intrapulmonary extracellular matrix and vascular basement membrane (11), thus directly leading to vascular permeability failure a leakage. In addition, MMP-9 enhances the chemotactic activity of neutrophils *via* the NF-κB pathway, increases the release of inflammatory factors, such as IL-6, and catalyzes the progression of pulmonary inflammation (12, 13). Second, the regulation of TF activation is associated with the disulfide bond transformation caused by redox imbalance (14). The thioredoxin system regulates oxidation-reduction balance. Thioredoxin (Trx)-1 interferes with FVIIa binding to purified and cell surface TF, thus suppressing TF-dependent procoagulant activity and proteinase-activated receptor-2 activation. Trx-1 also facilitates the reduction of TF, which causes a decrease in TF activity (15). Additionally, Trx-1 expression and MMP-9 activity are negatively correlated in cystathionine-β-synthase heterozygous mice; decreased Trx-1 causes an imbalance of NOX-4/TRX-1 enzymes, which lead to increased production of ROS and activation of MMP-9 (16). Therefore, targeting Trx-1 to inhibit TF expression and MMP-9 activity may be an effective way to reduce inflammatory cytokine storm damage and attenuate endotoxin-induced lung injury.

It was first reported that the inhalation of 2% hydrogen selectively neutralizes oxygen-free radicals to exert anti-oxidative stress and anti-inflammatory effects (17). Our previous studies showed that hydrogen improves central ischemia reperfusion and immune regulation (18, 19). Drinking saturated hydrogen water has a therapeutic effect on mice with interstitial pneumonia, and significantly reduces the release of IL-6 in plasma (20). Furthermore, many patients have experienced inflammatory cytokine storms during novel coronavirus infections (21). Professor Zhong Nanshan and colleagues proposed “inhalation of hydrogen and oxygen when possible,” which was included in the seventh edition of the “Guidelines on the Novel Coronavirus-infected Pneumonia Diagnosis and Treatment” issued by the National Health Commission of China. The proposal suggested that hydrogen plays a positive role in treating and relieving inflammatory lung injury among COVID-19 patients. In the current study we determined the effects of hydrogen on endotoxin-induced lung injury and the underlying mechanism. We report that hydrogen attenuates endotoxin-induced lung injury and increases the survival rate of endotoxin mice by upregulating Trx-1 expression to inhibit TF expression and MMP-9 activity.

## Materials and Methods

### Animals and Treatments

Male SPF-grade ICR mice, 6–8 weeks old and weighing 30 ± 5 g, were used in this study. Mice were provided by the Puer BHQ Laboratory Animals, Inc. (License Number: SCXK HU 2018-0006; Shanghai, China). The mice were housed in a controlled environment at 22 ± 2°C with a 12 h light-dark cycle (lights on at 8:00 a.m.) with access to food and water *ad libitum*. The mice were used after 7 days of adaptation to the environment. For each group of experiments, the animals were matched by age and body weight.

The model of endotoxin-induced ALI was induced by a single intraperitoneal (*i.p*.) injection of LPS (10 mg/kg). A total of 144 mice were randomly divided into four groups: control (*n* = 19); LPS (*n* = 42); LPS + H_2_ (*n* = 64); and single drug H_2_ (*n* = 19).

Considering the high mortality of endotoxemia, in order to determine the best time for hydrogen treatment, we referred to the previous literature (22–25) and firstly observed the 7-day survival rate of animals. Thirty-three mice were randomly selected from the hydrogen treatment group and divided into three groups: hydrogen treatment after 30 min of LPS (*n* = 11), hydrogen treatment after 6 h of LPS (*n* = 11) and hydrogen treatment after 12 h of LPS (*n* = 11). The survival rate of 7 days was observed together with the other three groups (11 mice in each group), and the best treatment time point was finally determined for subsequent experiments. In the following experiments, the LPS and LPS + H_2_ groups were randomly divided into three groups according to the following time points: 6 h (*n* = 9), 12 h (*n* = 17), and 24 h groups (*n* = 9). Eight mice were randomly selected from 12 h mice and divided into inhibitor model group and inhibitor treatment group.

The Hydrogen/Oxygen Generator was switched on 30 min in advance, and set with a hydrogen output pressure of 0.4 MPa and a flow rate of 400 ml/min. The laboratory animal box air was balanced to achieve a hydrogen concentration of 4%. LPS powder was dissolved in saline solution to obtain a concentration of 1 mg/ml. LPS was injected *i.p*. at a dose of 0.1 ml/10 g to obtain the endotoxemia model used in the LPS and LPS + H_2_ groups. The animals in the LPS + H_2_ group were immediately placed in the hydrogen tank after injection, while the animals in the H_2_ group were placed directly into the hydrogen tank and subjected to a continuous inhalation of hydrogen for > 8 h per day. Mice in the inhibitor group were intraperitoneally injected with PX12 (12 mg/kg) 6 h before LPS injection.

### Chemicals and Reagents

Hydrogen gas (4%) was produced using a Hydrogen/Oxygen Generator (model SPE-600; Jinan Haowei Experimental Instrument Co., Ltd., Jinan, China). Isoflurane (Webio) was purchased from the Beijing Keyue Huacheng Science and Technology Co., Ltd. (Beijing, China). Pentobarbital was purchased from Shanghai Yuyan Instruments Co., Ltd. (Shanghai, China). Lipopolysaccharide *Escherichia coli* O55:B5 was purchased from Sigma (St. Louis, MO, USA). Antibodies against Trx-1 were purchased from Cell Signaling Technology, Inc. (Beverly, MA, USA). Antibodies against MMP-9 and transferrin were purchased from Abcam (Cambridge, CB2 0AX, UK). Antibodies against TF were purchased from Santa Cruz Biotechnology, Inc. (Santa Cruz, CA, USA) and the protein ladder were purchased from Thermo (Waltham, MA, USA). PX_12_ was purchased from MedChemExpress (Monmouth Junction, NJ, USA). Antibodies against CD11b, CD45, and Ly-6G were purchased from Biolegend (San Diego, CA, USA). Antibodies against CD31 was purchased from Abcam (Anti-CD31 antibody ab28364,UK). Erythrocyte lysate was purchased from Solarbio (Beijing, China). RPMI-1640 and low-glucose DMEM cell culture media were purchased from Gibco (Waltham, MA, USA). Fetal bovine serum was purchased from Biological Industries (Kibbutz Beit Haemek, Israel). Endothelial cell growth factor (ECGS) was purchased from Shanghai Zhong Qiao Xin Zhou Biotechnology Co., Ltd. (Shangai, China). A double-sandwich ELISA kit was purchased from Proteintech (Rosemont, IL USA).

### Cell Lines for Experiment

Human mononuclear macrophages (THP-1, No. ZQ 0086) were purchased from the Shanghai Zhongqiao Biological Research Institute (Shanghai, China). Human umbilical vein endothelial cells (HUVEC-C, Product Number: 16-150828) were purchased from Jiangsu KeyGEN BioTECH Corp., Ltd. (Jiangsu, China). Both cell lines were purchased with an evaluation certificate.

### Histologic Analysis

For the histologic analysis, the mice were anesthetized with 0.3% pentobarbital, then the lung tissues were quickly collected. The samples were fixed in 10% formalin for 24 h, dehydrated in a series of graded ethanol, and embedded in paraffin. Then, microtome sections (5 μm) were cut and stained with hematoxylin and eosin, and evaluated using immunohistochemical and immunofluorescence techniques.

### Ultrasonic Testing

The mice were anesthetized with 2% sodium pentobarbital and ultrasonic testing was performed using a US PHILIPS EPIQ5 ultrasonic machine and L ultrasonic probe. The scanning frequency of the probe was 3.0–12.0 MHZ. Two dimensional/color Doppler flow imaging, pulse Doppler and continuous Doppler were performed, and heart rate was recorded simultaneously. The reflux signal and the image with clear contour of regurgitation spectrum were selected for analysis. The degree of reflux was observed, the length and velocity of reflux beam were measured, and the blood flow velocity and heart rate were recorded according to the appearance and duration of reflux spectrum. The test method involved Lichtenstein DA double blue dot + PLAPS point detection, which checks the distribution of the B line and assesses the distribution area of the B line in the scanning area for semi-quantitative analysis (26–28). Scan the anterior and lateral parts of the left and right sides of the chest, from parasternal to axillary midline, from subclavian to upper edge of liver on the left side, and from subclavian to cardiac floor on the right side. To correct the measurement bias, a double-blind design was used during the ultrasonic examination. Neither the investigators nor the sonographers were aware of the type of the group details. Each mouse underwent an ultrasonic examination three times; the examination was repeated by two sonographers with > 3 years of experience. The average values of the two measurements were statistically analyzed.

### Flow Cytometry

A total of 100 μL of whole blood was collected, and 3 ml of red blood cell lysate was added. PBS was used to rinse the mixture twice, followed by the addition of Ly6G-FITC, CD11b-APC, and CD45-PE antibodies. PBS was used to wash the sample again, then the mixture was resuspended in 1 ml of 0.01 M PBS and subjected to flow cytometry analysis (FACS Verse; BD, New Jersey, Franklin Lakes, USA). The cells positive for all three antibodies were considered to be neutrophils (29–31).

### Cell Culture

Cells were divided into the following six groups: control (*n* = 3), LPS (*n* = 3), LPS + H_2_ (*n* = 3), H_2_ (*n* = 3), LPS + PX_12_ (*n* = 3), and LPS + H_2_ + PX_12_ (*n* = 3). Samples were collected at 6, 12, and 24 h from the control, LPS, LPS + H_2_, and H_2_ groups. Samples from the LPS + PX_12_ and LPS + H_2_ + PX_12_ groups were collected at 12 h. The cell incubator (Thermo 8000WJ; Waltham, MA, city, state, USA) was set to 5% CO_2_, 37°C, and a controlled humidity. The hydrogen cell incubator was set to 65% H_2_, 20% O_2_, 5% CO_2_, 37°C, and a controlled humidity (PH-1-A; Wuxi Puhebio Co. Ltd., WuXi, China). After cells were seeded into plates, the medium was replaced with serum-free medium before treatment. The inhibitor, PX_12_, was administered 6 h before LPS stimulation. The cells from the LPS + H_2_ group were immediately placed into the hydrogen cell incubator after LPS stimulation.

THP-1 monocytes were incubated at 100 ng/mL PMA (162 nM) for 48 h to differentiate into macrophages, and then the differentiated THP-1 macrophages were placed in the culture medium without PMA for 24 h. THP-1 cells were cultured in RPMI-1640 medium supplemented with 10% fetal bovine serum, 1% streptomycin mixed solution, and 50 μmol/L of β-mercaptoethanol. The cell density of each well was ~5 × 10^6^/ml.

HUVEC-C cells were cultured in F12K/MEM medium supplemented with 10% fetal bovine serum and 1% ECGS. The culture was placed in an incubator (Thermo 8000 WJ) with 5% CO_2_ at 37°C. The cells were digested with 0.25% trypsin-EDTA and resuspended at a ratio of 1:2.

### Cell Immunofluorescence Assay

HUVEC-C cells were incubated in a confocal dish. After 36 h, the serum was replaced with serum-free medium. The cells were processed according to the pre-set grouping. Twelve hours later, the cell culture medium was discarded and the cells were fixed with paraformaldehyde. Donkey serum was used for sealing, and the incubation was carried out overnight at 4°C in a refrigerator with the addition of primary antibody. The next day, the fluorescent secondary antibody and DAPI were added, and a laser scanning confocal microscope (Carl Zeiss LSM710; Jena, Germany) was used to observe the cells and shooting. Image-Pro Plus software was used for quantitative analysis of scanned pictures.

### Western Blotting

Lung tissue and serum samples were lysed with RIPA lysis buffer containing a phosphatase protease inhibitor and PMSF, while cell samples were lysed with IP cell lysis buffer. The BCA method was used to quantify the total proteins. Western blot samples were prepared by adding and heating one-fourth of the loading volume. Ten percent SDS-PAGE gels were prepared for the detection of TF and β-actin, and 15% SDS-PAGE gels were prepared for detection of Trx1. Twenty microgram of total protein were collected from each sample, and electrophoresis was performed using a Bio-Rad electrophoresis system, followed by membrane transfer, incubation of the membrane with primary and secondary antibody, and ECL photochemical staining. Digital images were obtained using a chemiluminescence apparatus, and used for statistical analysis performed by Quantity One software.

### Gelatin Zymography

Lung tissue and serum samples were lysed with RIPA lysis buffer containing a phosphatase proteinase inhibitor and PMSF. Plasma samples were diluted 20 times using the lysis solution, and MMP-9/2 samples were prepared by adding one-fourth of the loading volume. The separation gel (8 and 4% concentrated gels) were prepared, and 20-μl samples were loaded. The constant voltage was set at 80 V, and the electrophoresis was carried out for 130 min at 4°C. After elution, incubation, staining, and discoloration, imaging analysis was performed using a chronograph. Statistical analysis was performed using Quantity One software.

### Determination of Inflammatory Factors by ELISA

Serum samples were diluted at a 1:4 ratio. According to the instructions provided with the Proteintech kit, sample addition, incubation, washing, and developing were performed in this order. The correction wavelength was set at 630 nm. The optical density (OD value) of each well was measured at 450 nm with ELISA, and the IL-6 level was measured in the serum from each mouse.

### Statistical Analysis

Statistical analysis was performed using GraphPad Prism 7 statistical software. Image J software was used to quantify the gray value and the optical density. Image J Pro software was used for the fluorescence density analysis. Adobe IIIustrator CS4 software was used for mapping. Results are expressed as the mean ± SD. The survival rate was statistically analyzed using the survival curve and calibrated by a log-rank (Mantel-Cox) test. One-way ANOVA was combined with a Bonferroni multiple comparison test inter-group comparison. A *P* < 0.05 was considered statistically significant.

## Results

### Hydrogen Attenuates Endotoxin-Induced Lung Injury and Improves the Survival Rate in Mice

**Figure 1:**
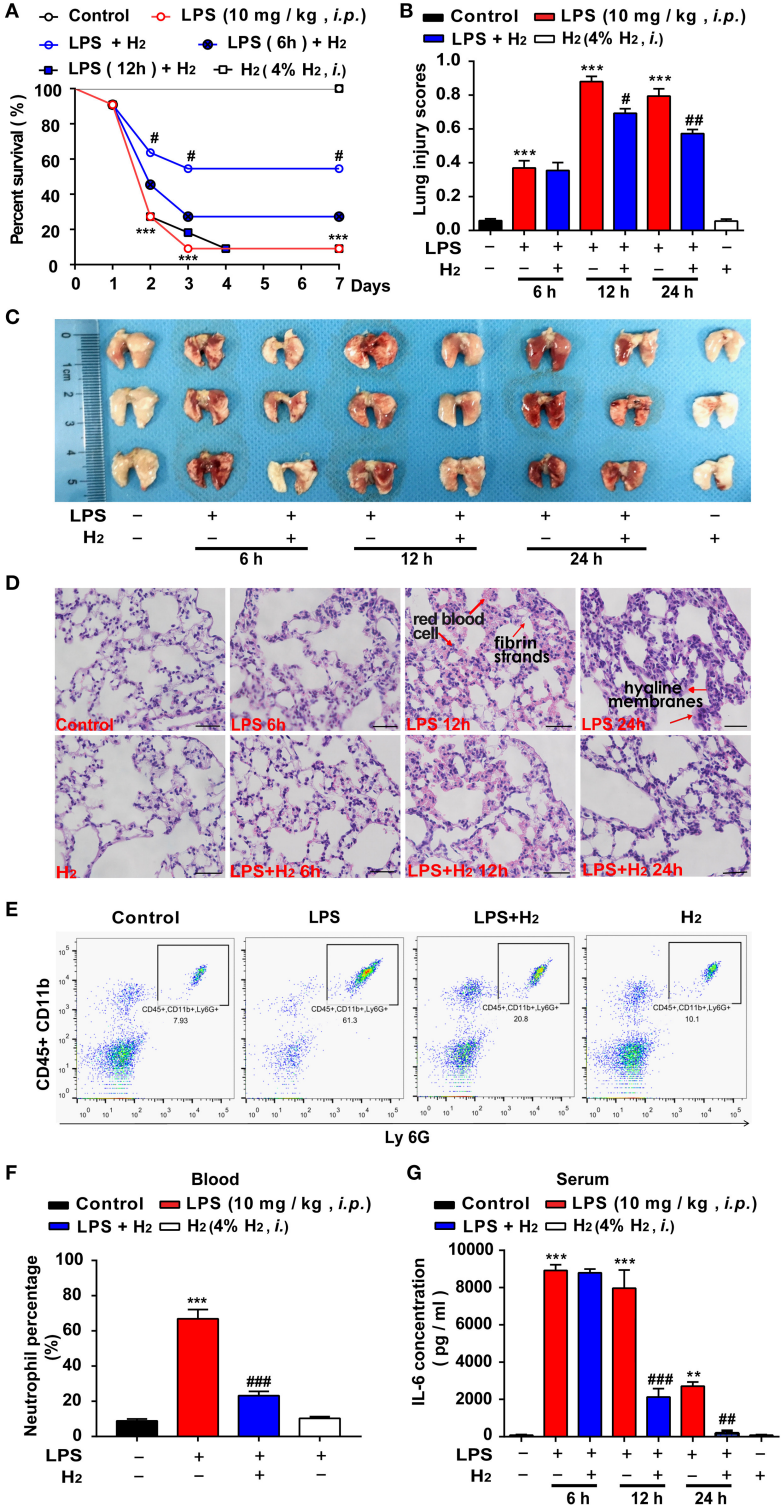
Hydrogen significantly alleviated LPS-induced acute lung injury. LPS (10 mg/kg *i.p*.) was used to establish endotoxemia mice. H_2_ (4% H_2_ inhalation) was used for treatment. The survival rate **(A)** of mice was measured every day for 7 consecutive days (*n* = 11 in each group). **(B)** Lung injury was significantly reduced at 12 and 24 h in the LPS + H_2_ group (*n* = 3 in each group). **(C)** Representative photographs of lung tissues after LPS injection at 6, 12, and 24 h (*n* = 3 in each group). **(D)** Hematoxylin- and eosin-stained sections of the lung tissues of mice at 6, 12, and 24 h. The “arrow” in the figure showed red blood cells, fibrin strands and hyaline membranes. Magnification: 400 × (*n* = 3 in each mice). Neutrophil proportion in blood **(E)** assessed by flow cytometric analysis and quantitative data of the neutrophil proportion **(F)** analyzed by flow cytometry at 12 h (*n* = 3 in each group). **(G)** IL-6 concentration in mouse plasma by ELISA at 6, 12, and 24 h (*n* = 3 in each group). A significant difference was revealed by one-way ANOVA (**P < 0.01, ***P < 0.001 vs. Control; ^#^*P* < 0.05, ^##^*P* < 0.01, ^###^*P* < 0.001 vs. LPS-treated group; Bonferroni *post-hoc* tests).

**Figure 2:**
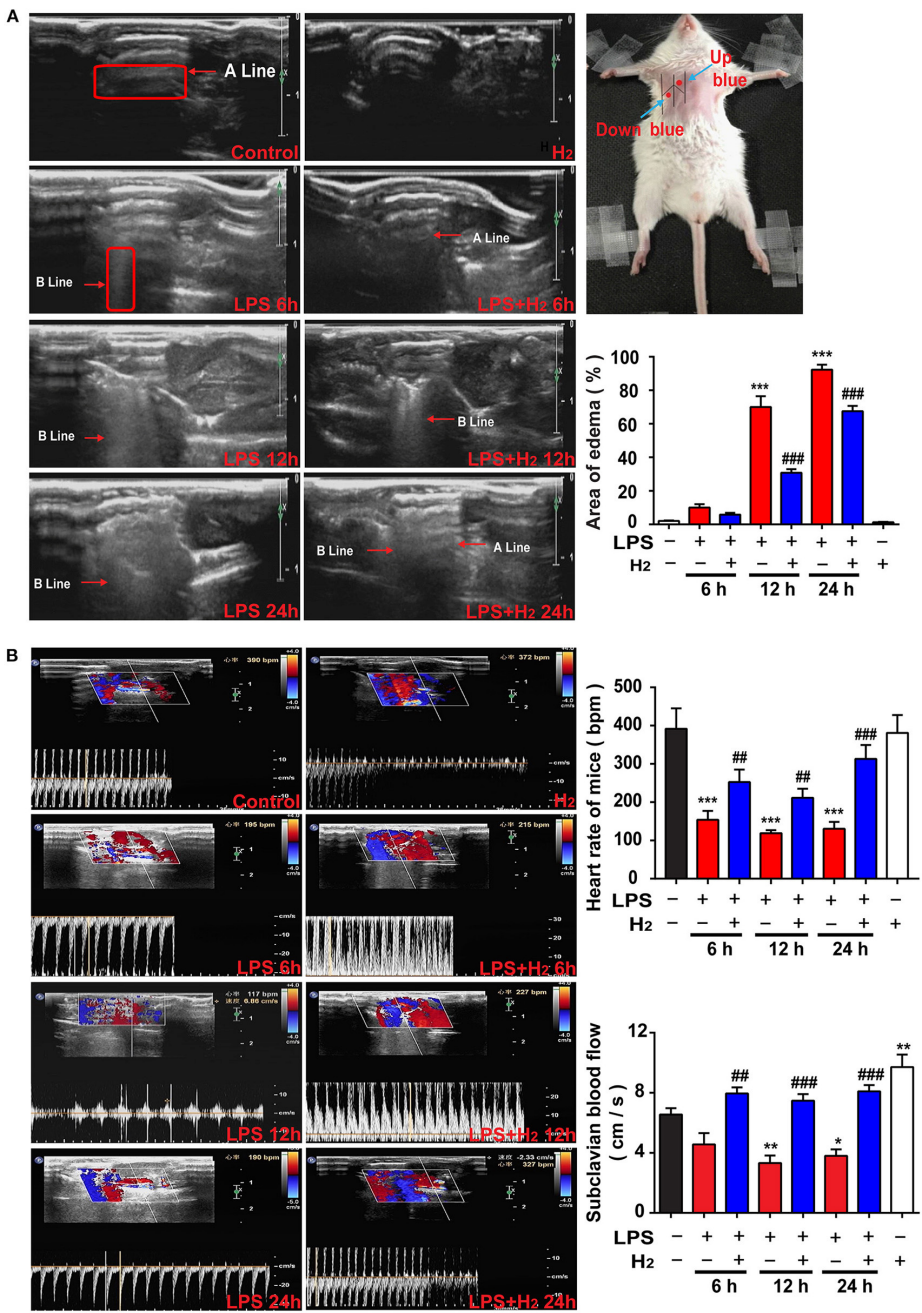
Hydrogen reduced endotoxemia-related pulmonary edema and improved the pulmonary circulation. LPS (10 mg/kg *i.p*.) was used to establish endotoxemia mice. H_2_ (4% H_2_ inhalation) was used for treatment. **(A)** Representative images of B-mode ultrasound. The “arrow” in the figure showed A line and B line. The pulmonary edema degree was assessed by the percentage of area scanned by the B line at 6, 12, and 24 h (*n* = 4 in each group). **(B)** Ultrasonic Doppler assessment on heart rates and blood flow velocity in the subclavian artery of mice at 6, 12, and 24 h (*n* = 4 in each group). A significant difference was revealed following one-way ANOVA (*P < 0.05, **P < 0.01, ***P < 0.001 vs. Control; ^##^*P* < 0.01, ^###^*P* < 0.001 vs. LPS-treated group; Bonferroni *post-hoc* tests).

First, we evaluated the effects of hydrogen on the survival rate of endotoxemia mice. As shown in Figure 1A, compared with LPS group, the survival rate of endotoxin mice treated with hydrogen at early stage was significantly improved. Hydrogen inhalation treatment after injection of LPS for 6 and 12 h did not show significant improvement. We further explored the protective effects of hydrogen on endotoxin-induced lung injury in endotoxemia mice. As shown in Figure 2B, compared with the LPS group, hydrogen reduced the acute lung hemorrhage, inflammatory cell infiltration, and alveolar septal thickening, resulting in a significant decrease in the lung injury score. The lung tissues of the mice were also observed after perfusion, and the lungs of mice in the control and H_2_ groups were shining and bright without blood stasis. Bleeding and swelling gradually increased in the lungs of the LPS model mice in a time-dependent manner (Figure 1C). A further pathologic examination of lung tissue sections showed that a small amount of red cell leakage was observed in the interstitial lungs at 6 h in the LPS group, large quantities of red cell exudate, inflammatory cell infiltration, alveolar collapse, and hyaline membrane formation were observed at 12 h, and a large number of inflammatory cells (mainly neutrophils) infiltrated the lung interstitium accompanied by extensive alveolar septal thickening at 24 h. In the H_2_ group, the lungs of mice showed a clear structure without expansion of the interstitial blood vessels or infiltration of inflammatory cells, the alveolar cavity was clean without exudation of red blood cells, and the alveolar wall was thin (Figure 1D).

Neutrophils in the blood were quantified at 12 h (Figure 1E). The results showed that neutrophils in the blood of endotoxemia mice were significantly increased at 12 h compared with the control group, while hydrogen markedly decreased the amount of neutrophils in the LPS + H_2_ group (Figure 1F). In addition, an ELISA showed that the concentration of IL-6 in the LPS group increased significantly at 6, 12, and 24 h. In the LPS + H_2_ group, hydrogen rapidly decreased IL-6 expression at 12 and 24 h (Figure 1G).

### Hydrogen Reduces Pulmonary Edema and Accelerates Pulmonary Circulation

Pulmonary edema was assessed by real-time ultrasound scanning. As shown in Figure 2, the lungs were filled with gas and the A line was clearly arranged in the control group. A high echogenic laser-like B line in the anterior chest wall and the area of the B line was one-third smaller than the scan area of the examining points were demonstrated in the LPS group at 6 h; a line could still be distinguished. At 12 h, the area of the B line was expanded between one-half and two-thirds, the A line disappeared, and the pulmonary edema was significantly aggravated. At 12 h, a diffuse B line fusion was observed in the lung that was almost entirely covered by scan points. The degree of edema in the lung of the LPS + H_2_ group at 6 h was similar to the LPS group. Of note, hydrogen decreased the area of the B line in the lung of LPS-treated mice at 12 h. The degree of diffusion of the B line in the LPS + H_2_ group was also significantly lower than the LPS group mice at 24 h, and the A line was vaguely identified in some lungs in the LPS + H_2_ group (Figure 2A).

During ultrasound scanning of the pulmonary edema, the Doppler mode was used to evaluate the blood flow velocity and heart rate of the subclavian artery in the mice. As shown in Figure 2B, compared with the control group, the blood flow velocity in the LPS group changed slightly at 6 h and slowed down significantly at 12 and 24 h. The LPS + H_2_ group showed an evident increase in blood flow velocity compared with the LPS group. Changes in heart rate and blood flow velocity were slightly different compared with the control group. Specifically, the LPS group showed a substantial decrease in heart rate at 6, 12, and 24 h. The heart rate of mice in the LPS + H_2_ group was significantly higher than the LPS group at 6, 12, and 24 h. In addition, hydrogen administration alone increased the blood flow velocity, but did not affect the heart rate of mice compared with the control group (Figure 2B).

### Hydrogen Inhibits TF Expression in the Lungs and Serum of Endotoxemia Mice

**Figure 3:**
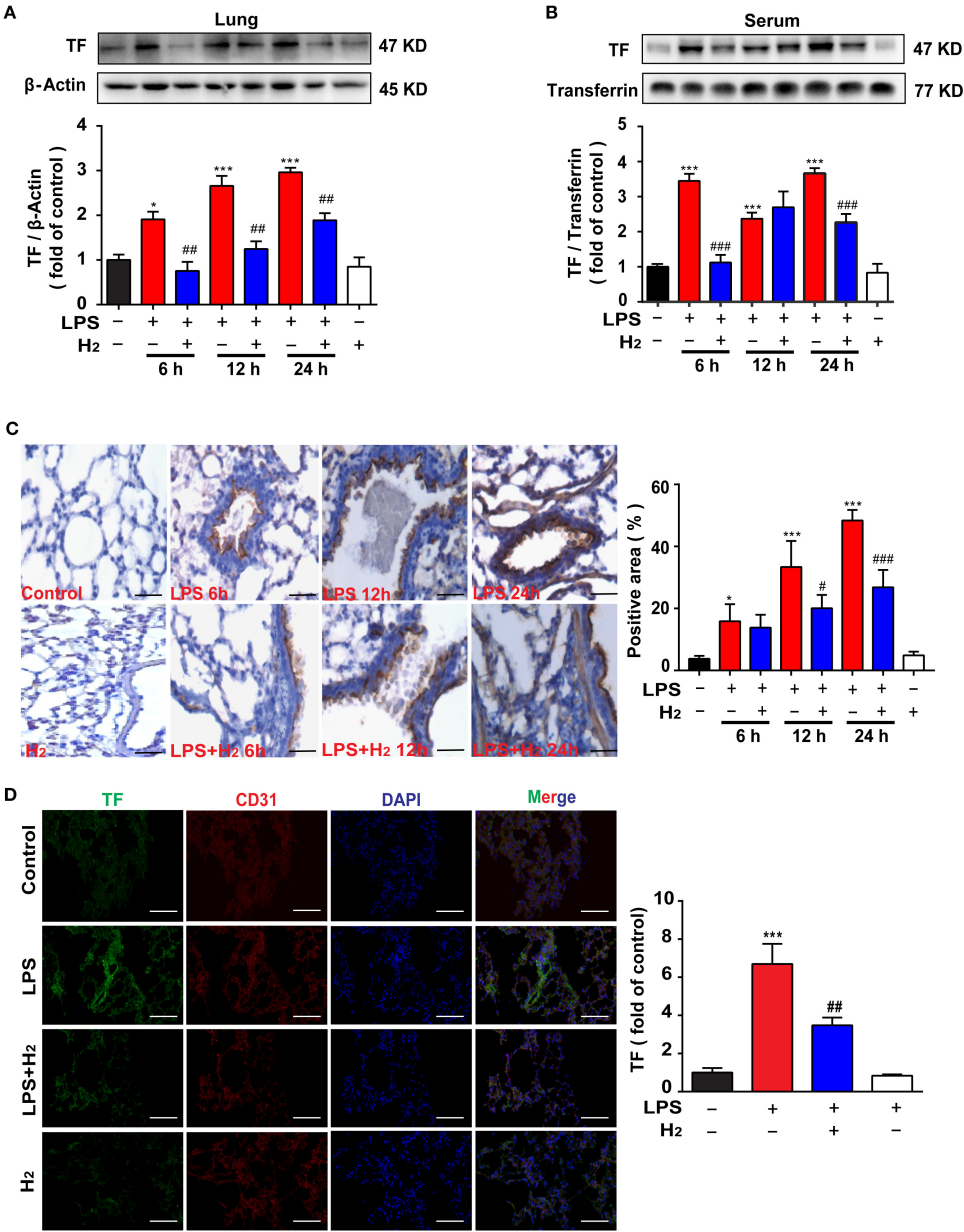
Hydrogen significantly suppressed LPS-induced increase of tissue factor (TF) in lungs and serum from mice. LPS (10 mg/kg, *i.p*.) was used to establish endotoxemia mice. H_2_ (4% H_2_ inhalation) was used for treatment. Effect of hydrogen on the expression of TF in lungs **(A)** and serum **(B)** were measured by western blot analysis and the densitometry values were normalized to β-actin and transferrin at 6, 12, and 24 h (*n* = 4 in each group). **(C)** Tissue paraffin sections of lungs subjected to TF immunohistochemical staining at 6, 12, and 24 h (*n* = 4 in each group). **(D)** TF expression by immunofluorescence detection in the lung tissues of mice at 12 h (*n* = 3 in each group). Significant difference was revealed following one-way ANOVA (*P < 0.05, ***P < 0.001 vs. Control; ^#^*P* < 0.05, ^##^*P* < 0.01, ^###^*P* < 0.001 vs. LPS-treated group; Bonferroni *post-hoc* tests).

The lung tissues and serum were used to evaluate the expression of TF. Compared with the control group, LPS significantly increased TF expression in lung tissues and serum at 6, 12, and 24 h, which were reduced by hydrogen (lung at 6, 12, and 24 h; serum at 6 and 24 h; Figures 3A,B). We further verified TF expression in lung tissues using immunohistochemical staining and obtained similar results (Figure 3C). LPS increased TF expression in lung tissues at 6, 12, and 24 h, which was decreased by hydrogen.

Furthermore, the lung slices at 12 h were selected for immunofluorescence staining. As shown in Figure 3D, compared with the control group, TF expression in the lung tissues of the LPS group was higher, while TF expression in the LPS + H_2_ group was significantly decreased by hydrogen (Figure 3D).

### Hydrogen Decreases MMP-9 Activity and Expression in the Lungs and Serum of Endotoxemia Mice

**Figure 4:**
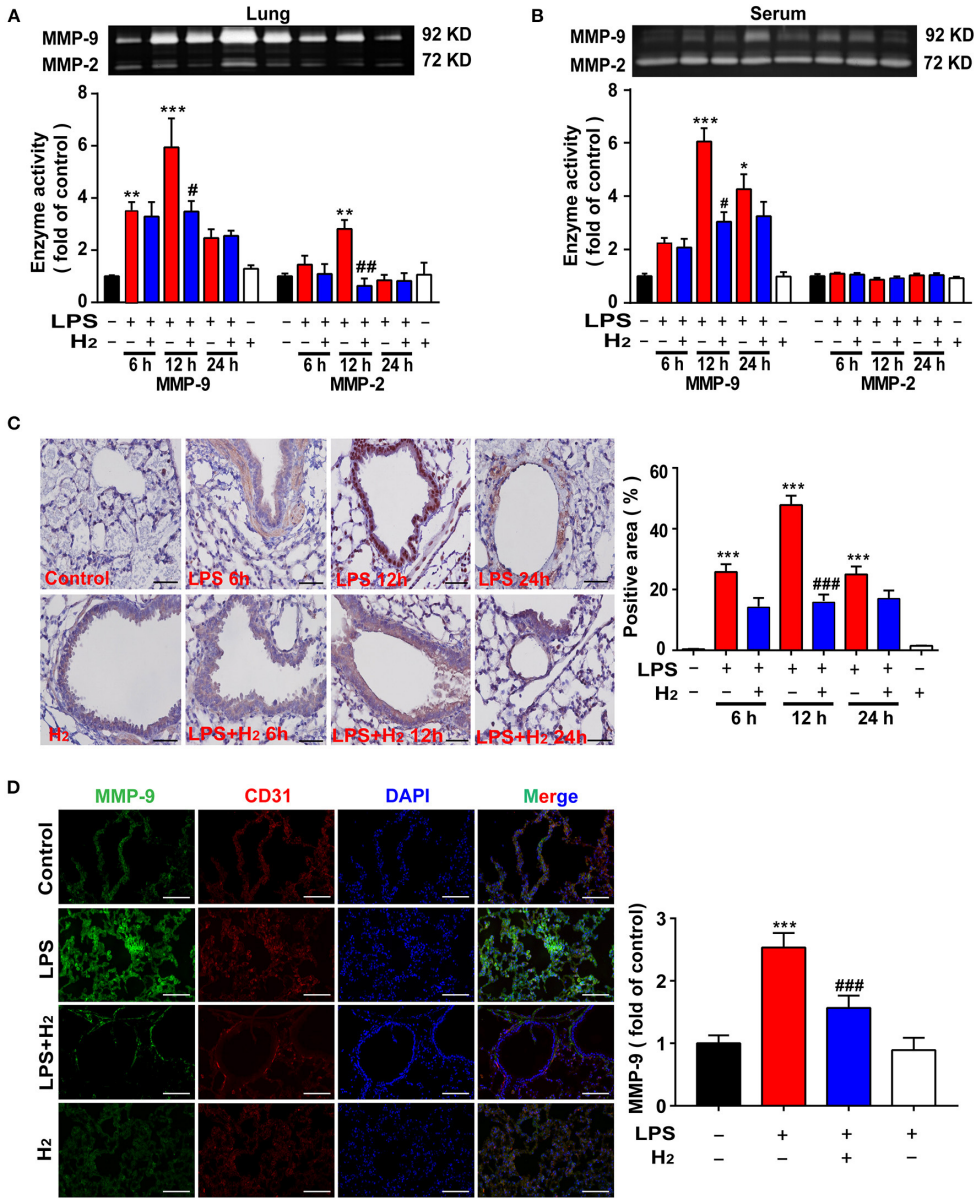
Hydrogen significantly suppressed LPS-induced increase of matrix metalloproteinase (MMP)-2/9 in lung and serum from mice. LPS (10 mg/kg, *i.p*.) was used to establish endotoxemia mice. H_2_ (4% H_2_ inhalation) was used for treatment. Effect of hydrogen on the expression of MMP-2/9 in lungs **(A)** and serum **(B)** were measured by gelatin zymography and the densitometry values were normalized at 6, 12, and 24 h (*n* = 4 in each group). **(C)** Tissue paraffin sections of lungs subjected to MMP-9 immunohistochemical staining at 6, 12, and 24 h (*n* = 4 in each group). **(D)** MMP-9 expression by immunofluorescence detection in the lung tissues of mice at 12 h (*n* = 3 in each group). Significant differences were revealed following one-way ANOVA (*P < 0.05, **P < 0.01, ***P < 0.001 vs. Control; ^#^*P* < 0.05, ^##^*P* < 0.01, ^###^*P* < 0.001 vs. LPS-treated group; Bonferroni *post-hoc* tests).

Gelatin zymography was carried out to quantify MMP-9/2 activity in the lung tissues and serum of mice. As shown in Figures 4A,B, LPS significantly increased MMP-9 activity in the lung tissues and serum at 6, 12, and 24 h compared with the control group. Inhalation of hydrogen gas significantly decreased MMP-9 activity both in the lungs and serum at 12 h.

We further verified the effects of hydrogen on MMP-9 using immunohistochemical staining. As shown in Figure 4C, LPS increased MMP-9 expression in the lung tissues at 6, 12, and 24 h compared with the control group, which was decreased by hydrogen. MMP-9 expression in the lungs by immunofluorescence staining was evaluated next at 12 h. The results showed that MMP-9 expression in the LPS group was higher, and MMP-9 co-localized with CD31 (a marker of vascular endothelial cells), suggesting that MMP-9 expression induced by LPS may be partly secreted from vascular endothelial cells. Hydrogen decreased MMP-9 expression markedly in the lung tissue (Figure 4D).

### Hydrogen Increases Trx1 Expression in Lung Tissues and Serum of Endotoxemia Mice

**Figure 5:**
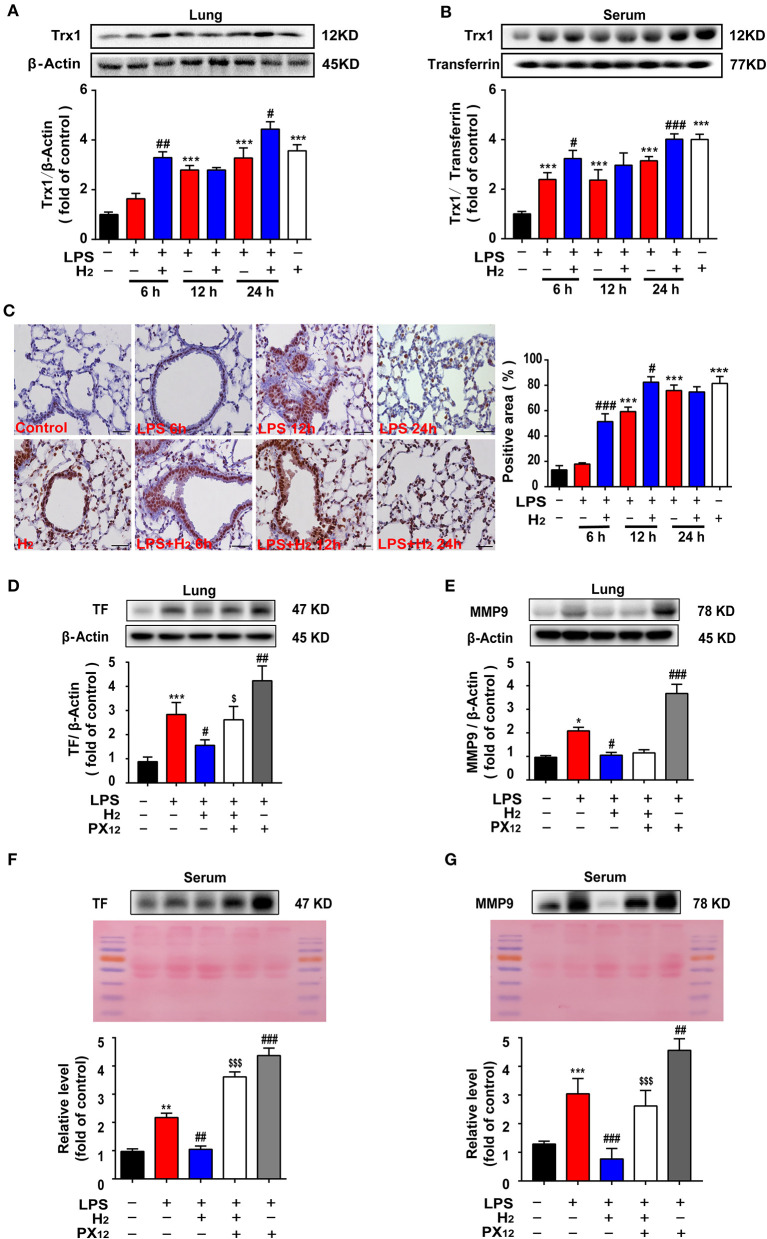
Hydrogen significantly upregulated the LPS-induced increase of Trx1 in lung and serum from mice. LPS (10 mg/kg, *i.p*.) was used to establish endotoxemia mice. H_2_ (4% H_2_ inhalation) was used for treatment. PX_12_ (12 mg/Kg) was intraperitoneally injected in mice as an inhibitor of Trx1. Effect of hydrogen on the expression of Trx1 in lungs **(A)** and serum **(B)** were measured by western blot analysis and the densitometry values were normalized to β-actin at 6, 12, and 24 h (*n* = 4 of each group). **(C)** Tissue paraffin sections of lungs subjected to Trx1 immunohistochemical staining at 6, 12, and 24 h (*n* = 4 of each group). Effect of hydrogen and PX_12_ on the expression of TF in lung **(D)** and serum **(F)** at 12 h were measured by western blot analysis and the densitometry values were normalized to β-actin (*n* = 4 in each group). Effect of hydrogen and PX12 on the expression of MMP-9 in lung **(E)** and serum **(G)** were measured by western blot analysis and the densitometry values were normalized at 12 h (*n* = 4 in each group). Significant differences were revealed following one-way ANOVA (*P < 0.05, **P < 0.01, ***P < 0.001 vs. Control; ^#^*P* < 0.05, ^##^*P* < 0.01, ^###^*P* < 0.001 vs. LPS-treated group; ^*$*^*P* < 0.05, ^*$$$*^*P* < 0.001 vs. LPS + H2 group; Bonferroni *post-hoc* tests).

We further explored the common targets for regulating TF and MMP-9. The lung tissues and serum were collected and Trx1 expression was evaluated. As shown in Figures 5A,B, hydrogen further increased Trx1 expression at 12 and 24 h in the lungs and serum of mice in the LPS + H_2_ group compared with the LPS group.

Immunohistochemical staining in the lungs was also observed at different time points; similar data were obtained (Figure 5C). LPS increased Trx1 expression in the lung tissues and hydrogen further increased Trx1 expression at 6, 12, and 24 h. Hydrogen administration alone significantly increased Trx1 expression compared with the control group.

In order to verify the effects of Trx1 on TF and MMP-9, mice were intraperitoneally injected with Trx1 inhibitor px12. Furthermore, as shown in Figures 5D–G, compared with the LPS group, hydrogen markedly decreased TF expression and MMP-9 activity induced by LPS in the lung tissues and serum, which were abolished by PX12, suggesting that hydrogen inhibited TF expression and MMP-9 activity *via* the Trx1-mediated signaling pathway.

### Hydrogen Inhibits TF Expression and MMP-9 Activity in HUVEC-C and THP-1 Cells *in vitro*

**Figure 6:**
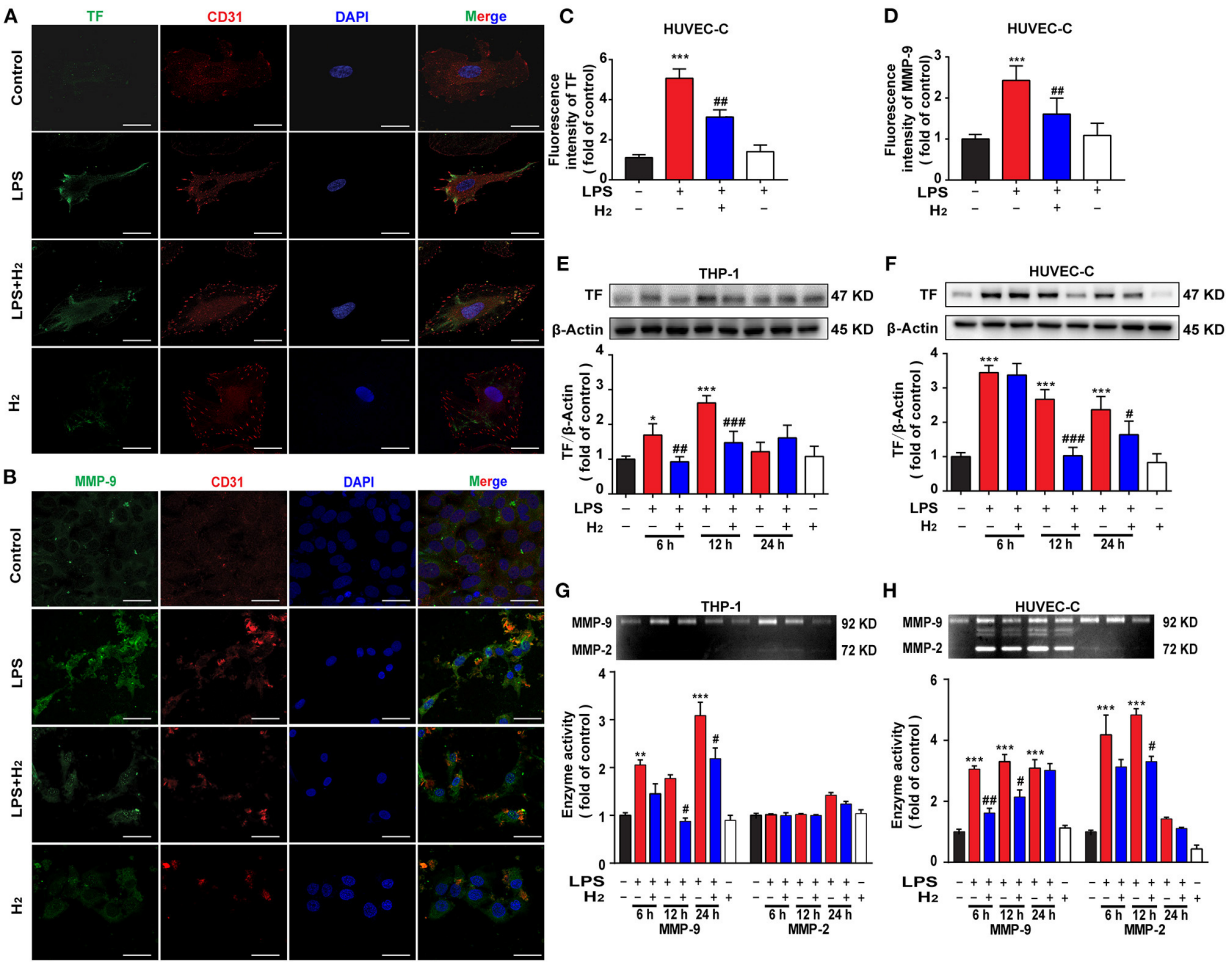
Hydrogen significantly suppressed the LPS-induced increase of tissue factor (TF) and matrix metalloproteinase (MMP)-2/9 in HUVEC-C and THP-1 cells. LPS (0.3 μg/ml) was used to establish endotoxemia model in HUVEC-C cells. LPS (1 μg/ml) was used to establish endotoxemia model in THP-1 cells. H_2_ (65% H_2_) was used to treat both HUVEC-C and THP-1 cells. TF **(A,C)** and MMP-9 **(B,D)** expression by immunofluorescence detection in HUVEC-C cells at 12 h (*n* = 3 in each group). Effect of hydrogen on the expression of TF in THP-1 **(E)** and HUVEC-C cells **(F)** were measured by western blot analysis and the densitometry values were normalized to β-actin at 6, 12, and 24 h (*n* = 3 in each group). Effect of hydrogen on the expression of MMP-2/9 in THP-1 **(G)** and HUVEC-C cells **(H)** were measured by gelatin zymography and the densitometry values were normalized at 6, 12, and 24 h (*n* = 3 in each group). Significant differences were revealed following one-way ANOVA (*P < 0.05, **P < 0.01, ***P < 0.001 vs. Control; ^#^*P* < 0.05, ^##^*P* < 0.01, ^###^*P* < 0.001 vs. LPS-treated group; Bonferroni *post-hoc* tests).

Based on the MTT-test results, the dose of LPS used to stimulate HUVEC-C and THP-1 cells was 0.3 and 1 μg/ml, respectively. HUVEC-C cells were used for the evaluation of TF and MMP-9 by immunofluorescence. As shown in Figures 6A,B, LPS significantly increased the expression of TF and MMP-9 in HUVEC-C cells 12 h after hydrogen exposure. Western blot results (Figures 6E,F) showed that LPS increased TF expression in THP-1 at 6 and 12 h and HUVEC-C cells at 6, 12, and 24 h. Hydrogen reduced TF expression in LPS-stimulated THP-1 cells at 6 and 12 h and HUVEC-C cells at 12 and 24 h.

We also measured MMP-9 activity *in vitro*. As shown in Figures 6G,H, LPS significantly increased MMP-9 activity in THP-1 and HUVEC-C cells in the control group at 6, 12, and 12 h. Hydrogen decreased MMP-9 activity in LPS-stimulated THP-1 cells at 12 and 24 h and in HUVEC-C cells at 6 and 12 h.

### Hydrogen Decreases TF Expression and MMP-9 Activity *via* the Trx1-Mediated Signaling Pathway in the HUVEC-C and THP-1 Cells *in vitro*

**Figure 7:**
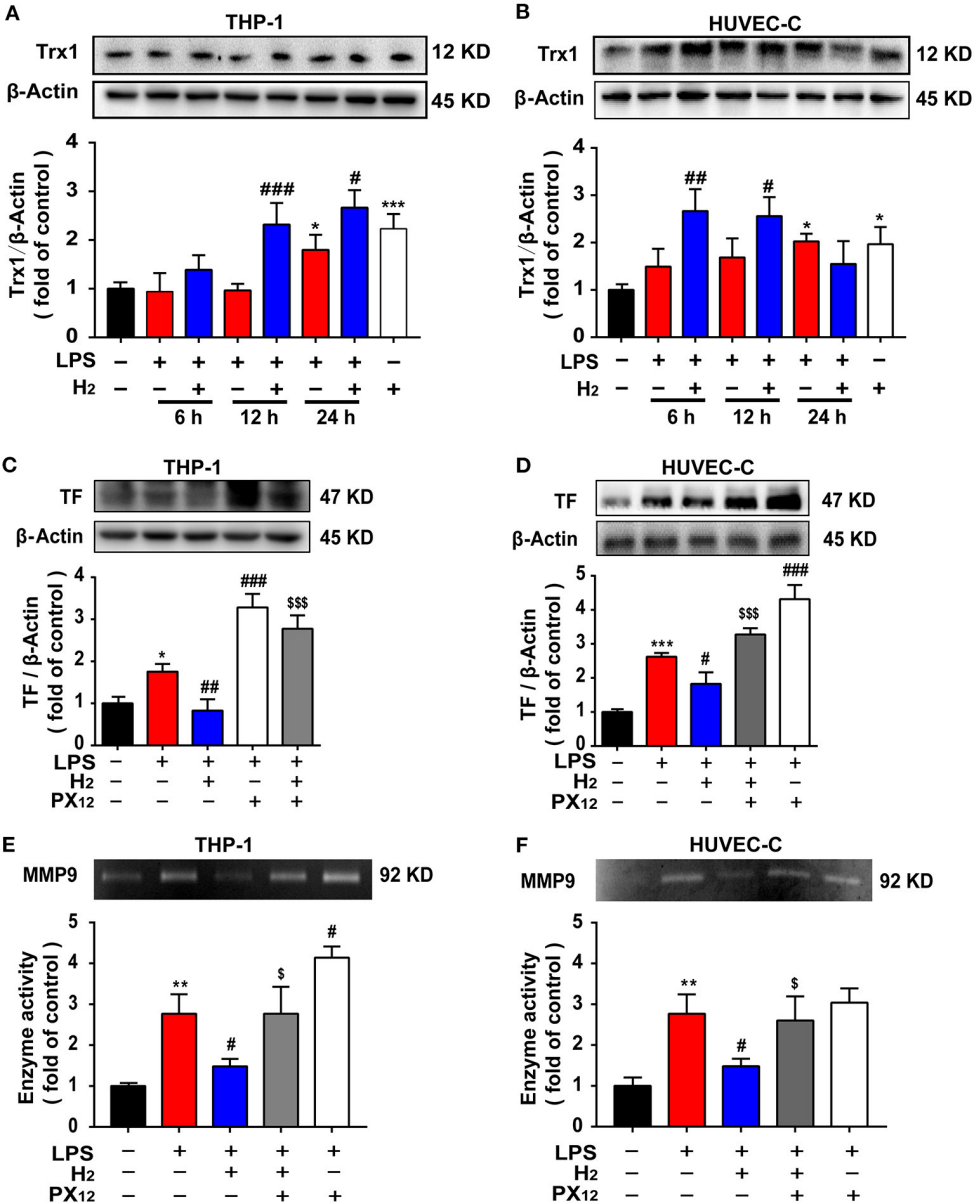
The expression of TF and MMP9 are closely related to Trx1. LPS (0.3 μg/ml) was used to establish the endotoxemia model in HUVEC-C cells. LPS (1 μg/ml) was used to establish the endotoxemia model in THP-1 cells. H_2_ (65% H_2_) was used to treat both HUVEC-C and THP-1 cells. PX_12_ (2 μM) was used in HUVEC-C cells as an inhibitor of Trx1. PX_12_ (8 μM) was used in THP-1 cells as an inhibitor of Trx1. Effect of hydrogen on the expression of Trx1 in THP-1 **(A)** and HUVEC-C cells **(B)** were measured by western blot analysis and the densitometry values were normalized to β-actin at 6, 12, and 24 h (*n* = 3 in each group). Effect of hydrogen and PX_12_ on the expression of TF in THP-1 **(C)** and HUVEC-C cells **(D)** at 12 h were measured by western blot analysis and the densitometry values were normalized to β-actin (*n* = 3 in each group). Effect of hydrogen and PX12 on the expression of MMP-9 in THP-1 **(E)** and HUVEC-C cells **(F)** were measured by gelatin zymography and the densitometry values were normalized at 12 h (*n* = 3 in each group). Significant differences were revealed following one-way ANOVA (*P < 0.05, **P < 0.01, ***P < 0.001 vs. Control; ^#^*P* < 0.05, ^##^*P* < 0.01, ^###^*P* < 0.001 vs. LPS-treated group; ^$^*P* < 0.05, ^$$$^*P* < 0.001 vs. LPS + H_2_ group; Bonferroni *post-hoc* tests).

We investigated the mechanism underlying hydrogen inhibition of TF and MMP-9 expression *in vitro*. As shown in Figures 7A,B, LPS increased Trx1 expression in THP-1 and in HUVEC-C cells at 24 h. Compared with the LPS group, hydrogen further increased Trx1 expression in LPS-stimulated THP-1 cells at 12 and 24 h and HUVEC-C cells at 6, 12, and 24 h.

To verify the effects of Trx1 on TF and MMP-9, the Trx1 inhibitor, PX_12_, was used in cell experiments. Furthermore, as shown in Figures 7C,D, compared with the LPS group, hydrogen markedly decreased TF expression and MMP-9 activity induced by LPS in THP-1 and HUVEC-C cells, which were abolished by PX_12_, suggesting that hydrogen inhibited TF expression and MMP-9 activity *via* the Trx1-mediated signaling pathway.

## Discussion

In this study, we showed the following major findings: (1) Inhalation of hydrogen gas significantly improved the survival rate of endotoxemia mice; (2) Inhalation of hydrogen gas reduced endotoxin-induced lung injury scores, decreased pulmonary edema, pulmonary hemorrhage, neutrophil infiltration, and IL-6 secretion, and accelerated the blood flow velocity of the pulmonary circulation; (3) Hydrogen decreased TF expression and MMP-9 activity, and increased Trx1 expression in the lungs and serum of endotoxemia mice; (4) Hydrogen decreased TF expression and MMP-9 activity in THP-1 and HUVECC cells induced by LPS, which were abolished by PX_12_; and (5) Hydrogen increased the expression of Trx1 in THP-1 and HUVECC cells.

We first evaluated the effects of hydrogen on endotoxin-induced lung injury. Inhalation of hydrogen gas significantly increased the survival rate of endotoxemia mice (Figure 1A), and improved lung injury and lung function, such as lung injury scores (Figure 1B), pulmonary edema and hemorrhage (Figures 1C, 2A), neutrophil infiltration (Figures 1D–F), IL-6 secretion (Figure 1G), and the blood flow velocity of the pulmonary circulation (Figure 2B). So, how does hydrogen attenuate endotoxin-induced lung injury?

Immune and coagulation system dysfunction are important causes in the pathophysiologic process of endotoxemia when TF is involved. Several exogenous stimuli, such as bacteria and oxidative stress products, promote macrophages and vascular endothelial cells to release TF, thus participating in the inflammatory storm and subsequent cascade reactions to aggravate lung injury. Current clinical studies and animal experiments have demonstrated that TF is one of the most valuable diagnostic biomarkers for severe endotoxemia, ARDS, and short-term mortality induced by endotoxemia (7, 32, 33). Thus, the overall level of TF expression was evaluated herein. LPS was successfully used *in vivo* to cause endotoxin-induced lung injury (Figures 1, 2) and death of mice (Figure 1). LPS was shown to be associated with the high expression of TF in the lung tissues and serum of mice at 6, 12, and 24 h after LPS stimulation (Figure 3). Similarly, LPS significantly increased the expression of TF in THP-1 and HUVEC-C cells *in vitro* (Figures 7C,D), indicating that a high level of TF was closely related to the pathologic process underlying endotoxin-induced lung injury. Next, we determined whether hydrogen regulates TF expression. As shown in Figures 4, 5A–F, hydrogen decreased TF expression in the lungs and serum of endotoxemia mice *in vivo* and in LPS-stimulated THP-1 and HUVEC-C cells *in vitro*, suggesting that in the early stage of endotoxemia hydrogen effectively protected endotoxemia mice by inhibiting TF expression.

The mechanism underlying hydrogen inhibition of TF expression was further investigated. TF depends on the cleavage of biological sites by other factors to exert its biological effect. The TF pathway inhibitor, TFPI, a physiologic inhibitor of TF, consists of a single-stranded glycoprotein that binds and inactivates TF (34). The MMP family, especially MMP-9, has a strong cleavage effect on TFPI, resulting in high accumulation of TF (35, 36). MMP-9 also has a cleavage effect on the ligand, CL8, that promotes the chemotaxis of neutrophils (37). In addition, MMP-9 acts as a gelatinase to hydrolyze the basement membrane and extracellular matrix of the blood vessels, directly impacting the structure of the respiratory tract and lungs (12, 38). Thus, MMP-9 activity was also measured in the current study. We found that MMP-9 activity in the lungs and sera of mice peaked 12 h after LPS administration (Figure 4). LPS stimulation increased MMP-9 activity in THP-1 and HUVEC-C cells *in vitro* at 6, 12, and 24 h (Figures 6G,H). After exposure to hydrogen, MMP-9 activity was significantly reduced both *in vivo* and *in vitro* (Figures 4, 6). The descending trend became most evident at 12 h compared to the LPS group. Therefore, we are of the opinion that hydrogen effectively inhibited MMP-9 expression, further reduced the cleavage effect on TF, neutrophil chemotaxis, and vascular basement membranes, and alleviated the inflammatory response.

In the early stage of endotoxemia, the thioredoxin system is one of the main forces regulating the oxidative stress balance. Trx1 promotes TF reduction and is negatively correlated with MMP-9 activity (39, 40). Our results showed that Trx1 expression in the serum increased rapidly 6 h after LPS injection (Figure 5B) and at 12 h in lung tissues (Figures 5A,C). Interestingly, hydrogen further increased the expression of Trx1 in the lungs and serum of endotoxemia mice, especially at 6 h (Figure 5). We further verified the effect of hydrogen on Trx1 *in vivo* and *in vitro*. Both *in vivo* and *in vitro* experiments found that compared with the LPS group, the expression of Trx1 was significantly higher in the hydrogen treatment group, and the activity of TF and MMP-9 was significantly inhibited. However, the inhibition of TF and MMP-9 *in vivo* and *in vitro* was abolished after using Trx1 inhibitor PX_12_ (Figures 5, 7). It has been reported that hydrogen has a good regulatory effect on excessive oxidative stress (41), and Trx1 is an important regulatory protein of oxidative stress *in vivo*. Therefore, we speculate that hydrogen exerts its anti-oxidative stress *via* regulation of Trx1 expression.

In the current study the ultrasonic assessment of pulmonary edema was used *in vivo* in animal experiments. In the fight against COVID-19 in China, clinicians also approved the use of this rapid, non-invasive method of detection (42). This method can replace the traditional method of evaluating lung edema with the dry-to-wet ratio, thus reducing the use of animals and alleviating animal pain. In addition, we did not explore the mechanism underlying the upregulation of Trx1 expression by hydrogen. Taken together with the findings from our previous study (43), we concluded that the upregulation of Trx1 expression is closely associated with activation of AMPK by H_2_, which will be investigated in corollary experiments.

Molecular hydrogen is a selective antioxidant with no evident side effects. Molecular hydrogen can quickly reach the target organ by crossing the biologic barrier. This study confirmed that in the early stage of endotoxemia, 4% hydrogen attenuated endotoxin-induced lung injury caused by the inflammatory storm and improved the survival rate of mice. The protective effect of hydrogen on lungs in our endotoxemia model was realized through the upregulation of Trx1 expression with inhibition of TF/MMP-9 activation and the inflammatory response. In the global outbreak of COVID-19, damage to multiple organs was observed caused by the inflammatory storm that became the core pathologic mechanism underlying critically ill patients with novel coronavirus pneumonia (NCP) (44). We have provided a potential approach to combat NCP in clinical practice by clarifying the mechanism underlying endotoxin-induced lung injury.

In summary, we demonstrated that hydrogen attenuated endotoxin-induced lung injury and improved the survival rate of endotoxemia mice by increasing Trx1 expression to decrease TF expression and MMP-9 activity. While endotoxemia does not exactly mimic clinical sepsis, our findings support the potential use of inhaled hydrogen in septic patients.

## Data Availability Statement

The raw data supporting the conclusions of this article will be made available by the authors, without undue reservation.

## Ethics Statement

The animal study was reviewed and approved by the Guide for the Care and Use of Laboratory Animals (Ministry of Science and Technology of China, 2006) and the Nanjing Medical University Animal Care and Use Committee (NMJU-ACUC).

## Author Contributions

QL, LH, JL, and PY performed the experiments and analyzed the results. QL, LH, BW, and WL carried out the animal experiments, Gelatin Zymography, and H&E staining. MX, YL, and WY carried out the Western blotting analysis, LJ, FH, and YS helped carry out the cell cultures. YL, MD, and WL conceived of the study, and participated in its design and coordination and helped to draft the manuscript. All authors read and approved the final manuscript.

## Conflict of Interest

The authors declare that the research was conducted in the absence of any commercial or financial relationships that could be construed as a potential conflict of interest.
